# Sirtuin Functions in Female Fertility: Possible Role in Oxidative Stress and Aging

**DOI:** 10.1155/2015/659687

**Published:** 2015-05-05

**Authors:** Carla Tatone, Giovanna Di Emidio, Maurizio Vitti, Michela Di Carlo, Silvano Santini, Anna Maria D'Alessandro, Stefano Falone, Fernanda Amicarelli

**Affiliations:** ^1^Department of Life, Health and Environmental Sciences, University of L'Aquila, Via Vetoio, 67100 L'Aquila, Italy; ^2^Infertility Service, San Salvatore Hospital, Via Vetoio, 67100 L'Aquila, Italy; ^3^Institute of Translational Pharmacology (IFT), National Council of Research (CNR), 67100 L'Aquila, Italy

## Abstract

In search for strategies aimed at preventing oxidative threat to female fertility, a possible role of sirtuins has emerged. Sirtuins (silent information regulator 2 (Sir2) proteins), NAD^+^ dependent enzymes with deacetylase and/or mono-ADP-ribosyltransferase activity, are emerging as key antiaging molecules and regulators in many diseases. Recently, a crucial role for SIRT1 and SIRT3, the main components of sirtuin family, as sensors and guardians of the redox state in oocytes, granulosa cells, and early embryos has emerged. In this context, the aim of the present review is to summarize current knowledge from research papers on the role of sirtuins in female fertility with particular emphasis on the impairment of SIRT1 signalling with oocyte aging. On this basis, the authors wish to build up a framework to promote research on the possible role of sirtuins as targets for future strategies for female fertility preservation.

## 1. Introduction

In humans the first aging phenotype is represented by the decline of female reproductive function. After maximum efficiency in the early 20s, fertility gradually decreases until menopause occurs at a mean age of 50-51 years [1]. This process becomes more dramatic in the late 30s, when, in spite of ovulatory cycles, fertility decline is manifested in increasing rates of infertility, miscarriage, and birth defects [1]. The same condition arises from* in vitro* fertilization (IVF) studies where female age is the most significant factor influencing clinical outcome. Certainly the biological clock regulating female reproductive lifespan is established in order to minimize pregnancy and birth delivery complications in advanced age [2] and to preserve energy for somatic maintenance [3]. Nevertheless, increasing postponement of the first pregnancy in most industrialized societies has been contributing to the wide spread of subfertility related to female aging [1, 4].

Although it is generally accepted that aging is a result of both inborn and environmental factors [5], gradual accumulation of damage by oxidative stress and its interplay with dicarbonyl stress has been considered the major mechanism underlying ovarian aging [6–8]. Beyond aging, oxidative stress may jeopardize ovarian function during endometriosis, metabolic syndromes, that is, diabetes and polycystic ovarian syndrome (PCOS), and anticancer therapies [9].

In search for strategies aimed at preventing oxidative threat to female fertility, a possible role of sirtuins has emerged. Sirtuins (silent information regulator 2 (Sir2) proteins) belong to a well conserved family of NAD^+^ dependent enzymes with deacetylase and/or mono-ADP-ribosyltransferase activity that have evolved to respond to a variety of stresses and are emerging as key antiaging molecules and regulators in many diseases [10, 11].

The aim of the present review is to summarize current knowledge on the role of sirtuins in the regulation of ovarian functions in an effort to identify common threads. On this basis, the authors wish to build up a framework to promote future research on the possible role of sirtuins as targets for female fertility preservation.

## 2. Ovarian Functions and Aging

Ovarian lifespan is the main determinant of female reproductive function. It provides a reserve of germ cells established prior to or soon after birth. Renewal potential of the follicle pool has been demonstrated in a limited number of species where neo-oogenesis in adult ovaries has been observed [12]. Independently of renewal, during reproductive lifespan follicle reserve gradually decreases. At puberty the gonad is endowed with 300.000 primordial follicles containing oocytes blocked at prophase of the first meiotic division (GV stage) that are periodically recruited into the growing phase and stimulated towards differentiation under the influence of regulators and pituitary hormones, which play the role of survival or death factors [13, 14]. As a result a few follicles reach the large antral follicle stage and complete meiosis until the metaphase II stage (MII) during ovulation to become competent for fertilization [15]. During folliculogenesis the oocyte undergoes a remarkable array of genetic, epigenetic, and cytoplasmic changes aimed at developing full competence for fertilization and production of normal offspring. This process relies on a continuous cross talk between oocytes and granulosa cells that ensure coordination of all the events orchestrated in the ovary under the influence of paracrine and endocrine factors [16]. Maintenance of ovarian reserve and development of oocyte competence at all stages can be hampered by extrinsic and intrinsic factors, which may target ovarian microenvironment during entire reproductive lifespan. Extrinsic factors include environmental insults, that is, xenobiotics and anticancer drugs [17]. Intrinsic factors may enclose ovarian or systemic diseases endometriosis, metabolic syndromes, that is, diabetes and PCOS, and ovarian aging [9].

This process is represented by gradual loss of quantity and quality of ovarian follicles. The size of oocyte/follicle pool declines exponentially with age, with a marked increase in the rate of disappearance from age of 37-38 years onwards. When the menopause is reached, the supply is reduced to a thousand or less follicles, a number insufficient to sustain the cyclic hormonal process necessary for menstruation [18]. Ovarian functional decline with aging is also characterized by reduced ability to produce oocytes competent for fertilization and further development (the so-called aged oocytes) [19]. In addition to several biological and molecular evidences, the central role of oocyte aging is proved by the observation that age-related decline in female fertility can be overcome by oocyte donation from younger women [20]. Aneuploidy, associated in most cases with spindle aberrations, disturbances in chromosome congression, mitochondrial alterations, and changes in gene and protein expression represent the most frequent phenotype of aged oocytes [19, 21].

Evolved from the “free radical theory of aging” [22], the oxidative stress theory of aging suggests that, as unavoidable by-products of metabolism, reactive oxygen species (ROS) are continuously generated within cells, mainly by the electron transport chain (ETC) of mitochondria. If these reactive chemical species are not efficiently scavenged, oxidative damage to biomolecules may occur [23]. More than a decade after the free radical theory of ovarian aging first proposed by Tarin [24], biological and clinical research has provided numerous evidence that increased ROS may contribute to follicular atresia and to oocyte aging in ovaries [25]. Oxidative stress generated by mitochondrial dysfunction is considered the main cause for telomere shortening, chromosomal segregation disorders, maturation and fertilization failures, or oocyte/embryo fragmentation [26, 27]. Looking for the aetiology of oxidative stress, a possible role could be ascribed to impaired follicular vascularization with aging [21, 28]. Factors contributing to perturbation of ovarian microenvironment are* advanced glycation end-products*. These factors may hamper ovarian stroma vessels, follicular growth, assembly of an efficient system of antioxidant enzymatic defence, and development of an efficient perifollicular vascularization [9].

Given their high reactivity and short half-life, measurement of ROS levels in the follicle microenvironment has led to conflicting results about their role in fertility [21]. In this regard, relevant findings are those by Lim and Luderer [29], who revealed significant age-related increases in oxidatively damaged lipids, proteins, and DNA in different ovarian compartments, including granulosa cells and ovarian interstitial tissue, along with alterations of antioxidant enzyme expression. Further evidence of oxidative stress in the ovarian follicle was obtained by research on stress signaling pathways in older granulosa cells [30, 31] and reduced ROS scavenging efficiencies in the follicular environment have been confirmed by observations in cumulus cells [32]. Enzymatic activity and protein level of superoxide dismutase (SOD), the enzyme that reacts with superoxide anion radicals to form oxygen and H_2_O_2_, were found to decrease with age, and lower levels of SOD activity are associated with unsuccessful IVF outcomes. Although the involvement of oxidative stress in ovarian aging is clear, there are poor evidences of possible benefits from antioxidant treatments in humans suggesting that further actors with a potential role in modulating redox balance in the ovary and its loss with aging need to be investigated [33].

## 3. Sirtuins

In the last 15 years, the complex process of cellular aging has been tightly linked to the action of sirtuins [34, 35]. Sirtuins are formerly known as class III nicotinamide adenine dinucleotide (NAD^+^) dependent histone deacetylases (HDACs), although they may use a variety of substrates that include structural proteins, metabolic enzymes, and histones [11, 36]. All sirtuins remove predominantly acetyl groups from cellular proteins, and this posttranslational chemical modification affects significantly protein localization and function. In this process, the acetyl group from the acetylated substrate is transferred to the ADP-ribose portion of NAD, releasing 2′-O-acetyl-ADP-ribose, nicotinamide, and the deacetylated substrate as products [37]. Since change in NAD^+^/NADH ratio controls the activity of sirtuins, all members of this family may have a crucial role in sensing the energetic status of the cell [38–40].

After the first discovery of the yeast ortholog silent information regulator 2 (Sir2) [41], sirtuins have been identified in prokaryotes and in metazoan, thus suggesting an evolutionary conserved requirement for these proteins in different organisms across phyla [42, 43]. To date, seven members of the sirtuin family have been identified in mammals (SIRT1-7) and each member has peculiar subcellular localization, function, and substrate specificity (for an extensive review, see [44]). Although the initial investigations on mammalian sirtuins focused mainly on SIRT1, as this member appears to be the closest mammalian homolog to yeast Sir2 [45], interest is growing in understanding the function of the related family members. Among SIRT1-7, all except SIRT4 exhibit deacetylase activity, whereas only ADP-ribosyltransferase catalytic activity has been demonstrated for SIRT4 [46]. SIRT1 and SIRT2 have been found in both the nucleus and cytosol; on the other hand, SIRT3, SIRT4, and SIRT5 have been found exclusively in mitochondria, while SIRT6 and SIRT7 have been localized only in the nuclear compartment [11, 46, 47].

Important evidence has been provided regarding the role of sirtuin-activated pathways in the regulation of age-related oxidative stress. Overexpression of Sir2 prevents the aged phenotype reported when* S. cerevisiae* is treated with H_2_O_2_ [48]. In addition, human fibroblasts treated with sublethal concentrations of hydrogen peroxide show cell cycle arrest, NAD^+^ depletion, and decreased sirtuin activity and accelerate cellular senescence [49].

## 4. SIRT1, a Key Regulator of Energy Metabolism and Oxidative Stress

Strong experimental evidence supports the notion that SIRT1 plays a crucial role in sensing and modulating the cellular redox status thus providing protective effects in cells and tissues exposed to oxidative stressors* in vitro* and* in vivo* [50–52]. SIRT1 is able to directly deacetylate key proteins involved in the cellular stress response, such as forkhead box O (FoxO) transcription factors. Evidence for the interaction between SIRT1 and FoxO was first provided by Brunet et al. [53]. van der Horst et al. [54] showed that mammalian SIRT1 was able to bind FOXO4, thus catalyzing its deacetylation in a NAD dependent manner, while Kobayashi et al. [55] demonstrated that FOXO4 activity was suppressed or enhanced by SIRT1 inhibitor or its activator, respectively. Later, many other authors observed that SIRT1-related cellular protection against oxidative stress can be achieved by upregulating key antioxidant enzymes, such as catalase (CAT), mitochondrial SOD (MnSOD), and peroxiredoxin, through forkhead box O- (FoxO-) dependent mechanisms [56–60].

In* in vitro* study, some authors established the fact that SIRT1 activates proliferator-activated receptor coactivator-1*α* (PGC-1*α*), maintaining its deacetylated active form into the nucleus, where it activates genes involved in a variety of biological processes and responses, including antioxidative protection, mitochondrial biogenesis, glucose/fatty acid metabolism, and oxidative phosphorylation (OXPHOS) [61, 62]. Increases in NAD^+^ levels due to environmental or endogenous factors may be sensed by SIRT1 that, by deacetylating the transcriptional coactivator PGC-1*α* at promoter regions, induces the expression of specific genes whose protein products can maintain the bioenergetic state of the cell [63]. SIRT1-mediated control of PGC-1*α* activity is virtually able to regulate the expression of oxidative stress genes, including glutathione peroxidase (GPx1), CAT, and MnSOD [64]. In muscle, SIRT1 expression is decreased in response to a high fat diet, thus promoting hyperacetylation and inactivation of PGC-1*α*, which in turn results in lower expression of PGC-1*α*-targeted genes involved in mitochondrial biogenesis [63].

The nuclear factor *κ*B (NF-*κ*B), which is a major inducer of inflammatory responses, was the first eukaryotic transcription factor described to respond directly to H_2_O_2_-induced oxidative stress [65]. NF-*κ*B deacetylated and inactivated by SIRT1 exhibits impaired downstream signalling and lowers the cellular ROS load by promoting the resolution of inflammation [66, 67].

Several experiments demonstrated that SIRT1 prevents replicative senescence in mammalian cells [68–70]. Conversely, the selective knockdown of SIRT1 at early passage was found to slow down cell growth, thus accelerating significantly cellular senescence [68]. Xu et al. [71] provided surprising evidence that senescence-promoting effects elicited by miR-22 in cancer cells are in part mediated by SIRT1. Further studies have clearly showed that the activity of SIRT1 can be fine-tuned by its own expression levels that are under the influence of several miRNAs known to suppress Sirt1 mRNA translation or reduce its stability [72–74].

As regards* ex vivo* studies, some authors have described a marked age-dependent decline in SIRT1 activity within rat liver, heart, kidney, lung, muscles, and cerebellum [75–77]. Moreover, our group found that SIRT1 expression decreases as a function of age in hippocampal formation of CD-1 mice [78]. It is also known that the SIRT1 protein levels decline in several murine disease or accelerated aging models [79, 80]. Conversely, both fasting and caloric restriction (CR) seem to induce SIRT1, in association with deacetylation and activation of PGC-1*α* [81]. Mice fed with a natural SIRT1 activator (e.g., resveratrol) exhibited significantly increased SIRT1 expression, as well as PGC-1*α* activity, enhanced mitochondrial biogenesis, and higher expression of the slow, oxidative myogenic program [82, 83].

## 5. Sirtuins and Female Reproductive Functions

### 5.1. Sirtuins and Reproductive Physiology: Insights from Transgenic Mouse Models

The role of sirtuins in the regulation of fertility has emerged when mice carrying a null allele of Sir2a were generated [84]. In all strains, Sirt1 deficient mice have a small, weak phenotype, with significantly increased postnatal mortality rates. However, as reported by Coussens et al. [85], Sirt1 deficiency is pleiotropic and dependent on cell type and/or stage of development. Sirt1 deficient mice display a number of developmental defects, most noticeably, irregularly shaped eyes and defective cardiac septation. In mouse embryonic fibroblasts, Sirt1 deficiency promotes extension of replicative lifespan and survival following exposure to genotoxic stress. Female and male Sirt1-null mice are infertile although data on the effect of Sirt1 deficiency on reproductive phenotype are controversial probably because of influence of mouse genetic background [84–86]. Sirt1-null mouse infertility can be readily overcome by assisted reproductive technologies although crossing with wild type is more successful. By crossing female or male wild type and/or knockout problems in gamete interaction have emerged. Indeed Sirt1-null mice exhibit reduced testis development associated with altered expression of 85 genes involved in spermatogenesis and protein sumoylation. In these mice, sperm are immotile and abnormal with increased numbers of small round-shaped heads and elevated DNA damage. Sirt1-null females appear not to cycle efficiently through oestrous and ovulation does not occur although follicles develop normally, perhaps indicating hormonal defects. Finally the reduction of early embryo development may be taken as further evidence for ovarian dysfunction and compromised oocyte developmental potential. Insight from knockout strains has also revealed that Sirt1 genotype is essential for normal embryogenesis: null offspring was smaller than normal at birth and most died during the early postnatal period. The importance of fine regulation of SIRT1 expression in reproductive functions is highlighted by the observation that reproductive maturity was significantly delayed in transgenic mice overexpressing SIRT1 when compared to wild-type controls [87]. In addition to Sirt1, gene-knockout experiments have contributed to elucidation of the role of Sirt3 in the reproductive phenotype [88]. Sirt3 silencing negatively affects mitochondrial activity and basal ATP synthesis although no obvious phenotype is observed in Sirt3^−/−^ mice indicating that the basal metabolic state could stand this reduction. Phenotype might become apparent under certain stress conditions: whereas Sirt3^−/−^ mice were fertile, IVF and* in vitro* cultured Sirt3^−/−^ or Sirt3 siRNA-induced knockdown embryos were susceptible to developmental defects [89].

### 5.2. Sirtuins in Mammalian Granulosa Cells, Oocytes, and Embryos

The expression of sirtuins has been observed in mammalian ovaries, granulosa cells, oocytes, and embryos (Table 1) [77, 89–91]. In rat ovaries it has been demonstrated that rapamycin and its target, mTOR, may inhibit the transition from primordial to developing follicles and preserve the follicle pool reserve through a fine-tuning of SIRT1 signalling which involves downstream activation of SIRT6 [92, 93]. In the same animal model, Wang et al. [94] observed that caloric restriction has a beneficial effect on ovarian lifespan through suppression of mTOR and activation of SIRT1 signalling. By contrast in ovaries from high fat diet, downregulation of SIRT1 signalling was associated with accelerated ovarian development and follicle loss.

In mammalian granulosa cells (GCs), the oocyte companion cells, which play a main role in follicle differentiation under the influence of oocyte and ovarian paracrine factors, and endocrine molecules, the role of sirtuins has been so far poorly investigated. From studies on SIRT1 and SIRT3, a possible role of this sirtuin in the regulation of proliferation and hormonal metabolism has emerged.

FOXL2 is a transcription factor belonging to forkhead box superfamily, regulating a number of biological processes such as development, differentiation, and proliferation, and is required for the normal development of the GCs [95]. In human granulosa cell lines KGN and COV434, it has been found that SIRT1-FOXL2 axis plays a key role in the maintenance of cell homeostasis [96]. This conclusion has arisen from the observation that SIRT1 inhibition by nicotinamide limits proliferation by promoting G1 arrest and DNA repair through dose-dependent deacetylation of FOXL2. Recent data by Pavlová et al. [97] in porcine GCs have revealed that SIRT1 is shown to stimulate cell proliferation by promoting accumulation of cyclin B1 and Cdc2/p34. In addition the interrelationships among SIRT1, NF-*κ*B/p50, and NF-*κ*B/p65 in transfected cells are relevant for control of proliferation and secretory activity of porcine GCs. In rat GCs overexpressing p50 and p65 and cultured in presence of increasing concentration of FSH, it has been reported that FSH promotes accumulation of both SIRT1 and NF-*κ*B. Therefore the SIRT1/NF-*κ*B system mediates FSH activity on progesterone and IGF-1 release, although it has been observed as a negative feedback control of FSH-induced NF-*κ*B by high amount of SIRT1 [98]. The SIRT1/NF-*κ*B system has also been demonstrated to be strictly connected with p53 and the mTOR; thus granulosa cell fate depends on the balance between inhibitory and stimulatory influences on SIRT1 [99].

Although it is recognized that resveratrol is an indirect and nonspecific activator of SIRT1, it has been employed to show that SIRT1 may play a key role in the activation of steroidogenesis associated with luteinization, the terminal differentiation of rat GCs [98]. Therefore the stimulation of SIRT1 by resveratrol would be potentially beneficial in the treatment of luteal phase deficiency. These observations provide evidence for direct involvement of SIRT1 in upregulation of ovarian hormone secretion. Finally, studies in human GCs and KGN cells show that SIRT1 signalling is involved in the response of ovarian cells to the insulin sensitizer metformin (MetF). MetF is known to increase NAD^+^/NADH ratio and SIRT1 abundance and activity in a dose-dependent manner [100, 101]. It has been recently demonstrated that MetF action on SIRT1 influences the expression of visfatin, a cytokine hormone and rate-limiting enzyme in NAD biosynthesis involved in metabolic (obesity, type II diabetes) and immune disorders [102].

Probably by acting as a sensor and regulator of ROS levels and mitochondrial functions, SIRT3 has been recently found to exert a positive role in the folliculogenesis and luteinization processes in GCs. Indeed, SIRT3 targets mitochondrial enzymes, such as glutamate dehydrogenase (GDH), and increases oxidative phosphorylation by deacetylation of enzymes involved in the electron transport chain [103]. In human granulosa and cumulus cells, a decrease has been reported in SIRT3 expression which is dependent on maternal age and ovarian reserve. This reduction has been associated with a lower active deacetylated GDH form, which contributes to the altered metabolism of follicle cells during aging [104]. Finally, SIRT3 depletion resulted in downregulation of steroidogenic enzymes and thus resulted in decreased progesterone secretion in human GC and COV434 cell line [105].

All sirtuin members are expressed in mouse ovulated MII oocytes, and their expression gradually decreases upon fertilization until the blastocyst stage [89].

In mouse GV and MII oocytes, SIRT1 expression has been associated with changes in chromatin configuration [90], oxidative stress response, reproductive aging [91], and postovulatory aging [77].

Since the acetylation status of proteins in oocytes is positively or negatively associated with oocyte aging [106], sirtuin deacetylation activity of histonic and non-histonic targets has recently been investigated in oocytes at different meiotic stages [90].

By changing its intracellular localization, activating GV chromatin rearrangement, and modulating antioxidant enzymatic response, SIRT1 has been shown to orchestrate the adaptive response to oxidative stress in mouse oocytes [91]. Further, SIRT1 is supposed to be relevant to oogenesis rather than fertilization and early embryo development where a relevant role of SIRT3 has arisen [89]. Indeed we observed reduced levels of Sirt1 transcripts in MII oocytes when compared with GV [91]. This is not surprising since degradation of specific transcripts is known to occur during completion of meiosis; thus SIRT1 reduction in MII can be taken as an evidence of the minor role of SIRT1 in postfertilization events [107].

Experimental evidence in mouse oocytes demonstrated the role of redox signalling in the regulation of SIRT1 [91]. Indeed in oocytes arrested at GV stage the gene transcript encoding SIRT1 is induced in response to* in vitro* exposure to H_2_O_2_. Although the transcriptional regulatory program controlling this process has not been delineated, we can speculate that SIRT1 as a chromatin-regulating factor participates in the changes of transcriptional activity triggered by oxidative stress.

Manipulating SIRT1 activity by means of a specific inhibitor Ex527, SIRT1 downstream pathway appears to rely on the activity of FOXO3A leading to upregulation of MnSod gene and prevention of ROS increase. A novel aspect of the adaptive response to oxidative stress in mammalian oocytes is the finding of an oxidative responsive miRNA. Given the regulatory role of several miRNAs in SIRT1 posttranscriptional regulation [72] and stress response [73], in the mouse oocyte, the indirect correlations between the expression levels of miR-132 and Sirt1 along with the observation that Sirt1 is a validated target of miR-132 strongly support the hypothesis that this microRNA is implicated in Sirt1 mRNA modulation [72]. Finally, the crucial role of SIRT1-FOXO3A-MnSOD pathway under regulation of miR-132 is confirmed by its disruption in aged oocytes showing a lower ability to regulate the Sirt1 gene and miR-132 levels in response to oxidative stress [91]. Inhibition of SIRT1 activity affected meiotic progression and caused a significant increase in ROS levels in concomitance with anomalies of spindle and chromosomal organization in* in vitro* matured oocytes. This suggests that SIRT1 activity protects the oocytes from oxidative damage caused by stress related to* in vitro* culture. Experiments in bovine oocytes have provided evidence that activation of SIRT1 in oocytes may be a potential countermeasure against age-associated events in oocytes derived from aged cows [108]. This conclusion is based on the findings that supplementation of maturation medium with N-acetyl-cysteine (NAC) reduced the levels of ROS in the oocytes independently of age while SIRT1 inhibition increased the ratio of abnormal fertilization [108].

As observed for SIRT1, SIRT2 enzymatic activity seems to be linked to fluctuations in cellular NAD levels [43]. A recent study has established the fact that SIRT2 mediates the acetylation status of tubulin in a NAD dependent manner [109]. In mouse oocytes, depletion of SIRT2 has been associated with increased spindle defects and chromosome disorganization and with impaired microtubule-kinetochore interaction [110]. In addition, in the same paper, the authors observed a lower SIRT2 protein level in oocytes from aged mice, suggesting that decreased SIRT2 may be a contributing factor to oocyte age-dependent deficits [110].

The role of sirtuins as upstream regulators of embryo gene transcription has emerged since 1994 when nicotinamide was found to inhibit mouse embryo development* in vitro* [111]. Nevertheless the interest in the potential role of sirtuins in reproductive physiology has been increasing in the last few years. According to this insight, studies have initially focused on sirtuin involvement in embryo development with particular interest in the role of SIRT3. According to Kawamura et al. [89], sirtuin inhibitors and siRNA-induced knockdown of Sirt3 promote ROS formation and decrease blastocyst formation indicating that SIRT3 acts as a protecting factor against stress conditions during* in vitro* fertilization and embryo culture by ensuring maintenance of mitochondrial functionality. An important role in SIRT3 signalling in mouse embryos is played by p53. Experiments in Sirt3-knockdown embryos have revealed that manipulation of SIRT3 activity results in changes of gene expression and ROS-p53 pathway. These observations in the mouse have been confirmed in porcine embryos where SIRT3 has been found to regulate early embryo development by modulating essential gene expressions in concert with SIRT1 and SIRT3 [112]. Thus targeting SIRT3 might be a potential measure for promoting positive outcome in IVF by improving developmental potential of early embryos.

## 6. The Role of Sirtuins in Fertility Promoting Strategies Based on Natural Compounds

In addition to extending lifespan in numerous invertebrates, natural and synthetic SIRT1 activating compounds (STACs) exert beneficial effects on age-related diseases (i.e., cancer, inflammation, cardiovascular diseases, and neurodegeneration), when administered by diet. Several classes of plant-derived metabolites such as flavones, stilbenes, chalcones, and anthocyanidins were shown to directly activate SIRT1* in vitro* [113, 114]. Most of the activators identified were polyphenolic with a structure-activity relationship characterized by planar multiphenyl rings bearing hydroxyl groups [114].

Resveratrol (3,5,4′-trihydroxystilbene) is the most potent* in vitro* natural SIRT1 activator. It was initially identified in 1940 as a phenolic substance in the white hellebore,* Veratrum grandiflorum*, a flowering plant, and later in grape wines [115]. Starting from natural STACs, the search for synthetic SIRT1 activators with greater potency, solubility, and bioavailability has rapidly advanced in recent years. Initially derivatives of an imidazothiazole scaffold were identified. They were chemically distinct from the polyphenol backbone of resveratrol but had the same KM lowering mechanism as resveratrol and a much lower EC_50_. Actually a more potent third generation of STACs based on benzimidazole and urea-based scaffolds was screened. However, as far as we know, resveratrol is the only STAC tested on mammalian fertility. A recent paper has provided evidence for the effectiveness of resveratrol in preventing ovarian aging in mice [116]. The authors demonstrated that administration of 7 mg/kg/die of resveratrol for 12 months improved fertility by extending ovarian lifespan, as evidenced by increased number and quality of ovulated oocytes, embryo developmental potential, and litter size. Although SIRT1 activity has not been assessed, increased Sirt1 mRNA levels were considered an indirect evidence for SIRT1 activation by resveratrol. Nevertheless, although it is accepted that STACs act by promoting SIRT1 activity* in vivo*, the mechanisms by which they activate this deacetylase were the subject of intense debate. Direct allosteric activation of SIRT1 through a lowering peptide substrate Km or indirect activation resulting from off-targets effects was proposed [114].

SIRT1 signalling has also been involved in the beneficial effects on fertility attained by dietary supplementation of compounds other than STACs. Indeed, young female mice receiving NAC in drinking water for 2 months presented increased rate of fertilized oocytes and early embryo development in association with higher expression level of Sirt1 and Sirt2 and increased telomerase activity length [116]. In another study, rats treated with 5 mg/kg/die of rapamycin in order to obtain a caloric restriction phenotype throughout mTOR suppression presented enhanced follicle reserve and increased expression of SIRT1 and SIRT6 [93]. On the other hand diet with deleterious effects on fertility results in Sirt1 suppression. This is the case of high fat diet that accelerated rate of follicle loss leading to premature ovarian failure (POF) by activating mTOR and suppressing SIRT1 signalling [94].

## 7. Conclusions and Future Directions

Our knowledge of sirtuins has grown exponentially over the last few years and has been uncovering the field of fertility for a few years. The majority of the work carried out so far on the role of sirtuins in reproductive functions has focused on SIRT1 and SIRT3, as the main redox regulators. As reported above, SIRT1 as the major nuclear deacetylase plays a pivotal role in the transcriptional response to changes in redox conditions and SIRT3, as the major mitochondrial deacetylase, acts as the* in situ* regulator of proteins which ameliorate damage in mitochondria, the major source of ROS in the cell. Oocytes and embryos are constantly challenged by stresses and privations and require adaptive responses for their survival. In addition to redox perturbations in the intraovarian microenvironment related to aging or diseases with an oxidative basis, reproductive cells have to face stress conditions during their manipulation during assisted reproductive procedure. In this scenario, current findings in mammalian oocytes and embryos fit well with the main roles of SIRT1 and SIRT3 established by previous work in somatic cells. SIRT1 is expressed at mRNA and protein level in ovarian and ovulated oocytes [90, 91] but is likely to play a primary role during oocyte stay in the ovary rather in fertilization event. Many evidences support the role of this sirtuin in the adaptive response to oxidative stress [91] as well as in protecting oocyte against loss of developmental competence with reproductive and postovulatory aging [77, 90]. The SIRT1-FOXO3A axis might be one of the signalling pathways with a main role in this process. Based on the observation of increased ROS levels and disturbed spindle organization under SIRT1 inhibition, a potential role of SIRT1 as a guardian of meiosis might be also suggested. When our attention moves to postfertilization events, maternally derived SIRT3 appears to be a critical protein in the protection of early embryos against stress conditions during* in vitro* fertilization and culture. It is essential in maintaining mitochondrial homeostasis SIRT3 thus preventing the activation of the ROS-p53 pathway responsible for developmental defects. Finally, possible role of SIRT1 and SIRT3 in folliculogenesis and luteinization processes in GCs has emerged, with SIRT1 involved as a sensor of nutritional status and regulator of cell cycle and SIRT3 acting as a guardian of the redox state and steroidogenic metabolism. In addition, the findings open direct or indirect modulation of sirtuin activity by nutritional interventions with beneficial effects on ovarian physiology opening new horizons to their potential clinical implication in fertility care.

## Figures and Tables

**Table 1:** Sirtuins and related functions in the ovary.

Sirtuin	Intracellular localization	Ovarian cell type	Species	Function	Mediator	References
		Ovary	*Rat *	Folliculogenesis	mTOR; FOXO3a; NRF-1; SIRT6	Luo et al. [92]; Zhang et al. [93]; Wang et al. [94]
			*Human *	Proliferation; activation of steroidogenesis	Visfatin	Reverchon et al. [102]
		Granulosa cells	*Porcine *	Proliferation; secretory activity	p53; NF-*κ*B; MAPK; ERK1-2	Pavlová et al. [97]; Sirotkin et al. [99]
			*Rat *	Mediation of FSH action; activation of steroidogenesis	StAR	Morita et al. [98]
SIRT1	Cytoplasmic Nuclear	KGN		Cell homeostasis; response to metformin; activation of steroidogenesis; proliferation	FOXL2; visfatin	Benayoun et al. [96]; Cantó et al. [100]; Caton et al. [101]; Reverchon et al. [102]
		COV434		Cell homeostasis	FOXL2	Benayoun et al. [96]
		Oocyte	*Mouse *	Chromatin configuration; maturation; oxidative stress response; aging process	FOXO3a; miR-132; SOD2	Kawamura et al. [89]; Manosalva and González [90]; Di Emidio et al. [91]
			*Porcine *	Maturation		Wang et al. [94]
		Embryo	*Porcine *	Embryo development; regulation of apoptosis		Kwak et al. [112]

SIRT2	Cytoplasmic Nuclear	Oocyte	*Mouse *	Metaphase II spindle assembly and chromosome alignment; aging process	Histone H4K16 and *α*-tubulin	Kawamura et al. [89]; Zhang et al. [110]
		Embryo	*Porcine *	Embryo development; regulation of apoptosis		Kwak et al. [112]

		Granulosa cells	*Human *	Follicle metabolism; aging process; folliculogenesis; luteinization; progesterone secretion; oxidative stress response	GDH; SOD1; CAT; 17*β*HSD1; StAR; P450arom	Pacella-Ince et al. [104]; Fu et al. [105]
		COV434		Folliculogenesis; luteinization; progesterone secretion	SOD1; CAT; 17*β*HSD1; StAR; P450arom	Fu et al. [105]
SIRT3	Mitochondrial	Cumulus cells	*Human *	Follicle metabolism; aging process	GDH	Pacella-Ince et al. [104]
		Oocyte	*Mouse *	Oxidative stress response; maintenance of mitochondrial functionality		Kawamura et al. [89]
		Embryos	*Mouse *	Embryo development; oxidative stress response; maintenance of mitochondrial functionality	p53	Kawamura et al. [89]
			*Porcine *	Embryo development; regulation of apoptosis; marker of embryo potential		Kwak et al. [112]

SIRT4	Mitochondrial	Oocyte	*Mouse *	Function not investigated		Kawamura et al. [89]

SIRT5	Mitochondrial	Oocyte	*Mouse *	Function not investigated		Kawamura et al. [89]

SIRT6	Nuclear	Ovary	*Rat *	Folliculogenesis	mTOR; FOXO3a; NRF-1; SIRT6	Luo et al. [92]; Zhang et al. [93]; Wang et al. [94]
		Oocyte	*Mouse *	Follicle development		Kawamura et al. [89]; Wang et al. [94]

SIRT7	Nuclear	Oocyte	*Mouse *	Function not investigated		Kawamura et al. [89]
